# Methicillin-Sensitive and Methicillin-Resistant *Staphylococcus aureus* Nasal Carriage in a Random Sample of Non-Hospitalized Adult Population in Northern Germany

**DOI:** 10.1371/journal.pone.0107937

**Published:** 2014-09-24

**Authors:** Jaishri Mehraj, Manas K. Akmatov, Julia Strömpl, Anja Gatzemeier, Franziska Layer, Guido Werner, Dietmar H. Pieper, Eva Medina, Wolfgang Witte, Frank Pessler, Gérard Krause

**Affiliations:** 1 Department of Epidemiology, Helmholtz Centre for Infection Research, Braunschweig, Germany; 2 Hanover Medical School, Hannover, Germany; 3 TWINCORE Centre for Experimental and Clinical Infection Research, Hannover, Germany; 4 Robert Koch Institute, Wernigerode, Germany; 5 Microbial Interactions and Processes Research Group, Helmholtz Centre for Infection Research, Braunschweig, Germany; 6 Infection Immunology Research Group, Helmholtz Centre for Infection Research, Braunschweig, Germany; University Hospital Münster, Germany

## Abstract

**Objective:**

The findings from truly randomized community-based studies on *Staphylococcus aureus* nasal colonization are scarce. Therefore we have examined point prevalence and risk factors of *S. aureus* nasal carriage in a non-hospitalized population of Braunschweig, northern Germany.

**Methods:**

A total of 2026 potential participants were randomly selected through the resident's registration office and invited by mail. They were requested to collect a nasal swab at home and return it by mail. *S. aureus* was identified by culture and PCR. Logistic regression was used to determine risk factors of *S. aureus* carriage.

**Results:**

Among the invitees, 405 individuals agreed to participate and 389 provided complete data which was included in the analysis. The median age of the participants was 49 years (IQR: 39–61) and 61% were females. *S. aureus* was isolated in 85 (21.9%; 95% CI: 18.0–26.2%) of the samples, five of which were MRSA (1.29%; 95% CI: 0.55–2.98%). In multiple logistic regression, male sex (OR = 3.50; 95% CI: 2.01–6.11) and presence of allergies (OR = 2.43; 95% CI: 1.39–4.24) were found to be associated with *S. aureus* nasal carriage. Fifty five different *spa* types were found, that clustered into nine distinct groups. MRSA belonged to the hospital-associated *spa* types t032 and t025 (corresponds to MLST CC 22), whereas MSSA *spa* types varied and mostly belonged to *spa*-CC 012 (corresponds to MLST CC 30), and *spa*-CC 084 (corresponds to MLST CC 15).

**Conclusion:**

This first point prevalence study of *S. aureus* in a non-hospitalized population of Germany revealed prevalence, consistent with other European countries and supports previous findings on male sex and allergies as risk factors of *S. aureus* carriage. The detection of hospital-associated MRSA *spa* types in the community indicates possible spread of these strains from hospitals into the community.

## Introduction

The increased awareness of community-acquired methicillin-resistant *Staphylococcus aureus* (MRSA) requires reliable data on the prevalence of *S. aureus* carriage in the general (non-hospitalized) population. Population based studies from North America and Europe indicate a prevalence of *S. aureus* between 18% and 30% [1]–[6]. Data on prevalence in the general population of Germany - a country with a comparatively intermediate MRSA incidence in hospitals are limited. In addition, data are mostly assessed for selected population groups and epidemiological information associated with *S. aureus* carriage is rare [7], [8]. We therefore examined point prevalence and risk factors of *S. aureus* nasal carriage in a random sample of the non-hospitalized population of Braunschweig, Germany, and compared the findings with other prevalence assessments of nasal *S. aureus* carriage in the general population in Europe and other parts of the world. A secondary objective was to evaluate molecular epidemiological data and antimicrobial resistance patterns of *S. aureus* strains in the general population.

## Methods

### Study area, data and sample collection

This is a community based study in the general population of Braunschweig, a city with 246, 742 inhabitants in the Lower Saxony, Germany [9]. In Germany, the residents' registration office of the city is a local authority where all citizens of that particular area are obliged to register their address. Approximately 2000 men and women, 20–69 years of age were randomly selected through the resident's registration office of Braunschweig and invited to participate in the study by mail, without taking any consideration of risk factors of infection or health care contact of participants. The study was planned as a feasibility study of the German National Cohort (GNC), therefore only adult individuals 20 to 69 years of age were recruited [10]. As aimed for GNC, the proportion of younger participants (20–29 years, 30–39 years) was 10% and for older participants (40–49 years, 50–59 years and 60–69 years) the proportion was 26.7% and equal sex distribution of participants was retained for recruitment in this study [10]. Study aims and objectives were explained using information flyers to the invited participants. Information about the study was also made available through a press release in the local newspapers and on the website of the Helmholtz Centre for Infection Research, Braunschweig. A total of 405 persons agreed to participate in the study. Participants were asked to provide a self-administered nasal swab each month over a period of six months. To estimate point prevalence, this analysis is based on the data from one time point, i.e. first nasal swab received from each participant. Details of the recruitment methods, non-responder analysis, and the feasibility of obtaining serial self-collected nasal swabs, are reported separately [11]. At recruitment, the study participants were also requested to complete a questionnaire containing information on basic demographic characteristics such as age, sex, and potential risk factors for methicillin-sensitive and methicillin-resistant *S. aureus* (MSSA and MRSA). The questionnaire items covered skin infection, outpatient clinic visits, hospital stay of at least one night, surgery, antibiotics prescribed, or international travel occurring in the last 12 months, as well as pet animal contact, occupational animal contact, presence of allergies, and presence of diabetes mellitus. In addition, detailed and illustrated instructions on how to collect a nasal swab were provided.

### Laboratory analysis

All swabs (Amies agar gel swabs 108C, Copan Diagnostics, Brescia, Italy) were inoculated on blood agar and in parallel on MRSA chrome agar plates (BD Diagnostics, Heidelberg, Germany). The plates were incubated at 37°C (Heraeus, Wehrheim, Germany). Cultures were not kept for more than 48 hours in the incubator. The appearance and growth score on blood agar were recorded. Colonies suspected to be *S. aureus* were characterized further with the slide coagulase test. Human blood plasma received from a local hospital and fibrinogen from human blood plasma (Sigma Aldrich, Inc. USA), were used for coagulase testing. DNA was extracted from colonies with DNeasy Blood and Tissue kit (Qiagen, Hilden, Germany). All *S. aureus* were subjected to PCR targeting the *spa* and *mec*A genes, using standard primers as described elsewhere [12], [13]. Amplification conditions consisted of 3 min at 94°C, followed by 30 cycles of 30 sec at 94°C, 30 sec at 55°C, and 30 sec at 72°C, with a final step of 4 min at 72°C. The DNA fragments were separated by gel electrophoresis on a 1.5% agarose gel (Invitrogen, Merekel, Belgium) stained with ethidium bromide.

PCR products were purified with the QIAquick Nucleotide Removal Kit (Qiagen, Hilden, Germany). Molecular typing was performed by means of *spa*-typing which is based on polymorphisms in the X-region of the *spa* gene. PCR amplification and sequencing of this region was performed as described previously [12]. The software Ridom StaphType (Ridom GmbH, Münster, Germany) was used for *spa* sequence analysis and attribution to types [14]. Based Upon Repeat Pattern (BURP) algorithm was used to define *spa* clonal complexes (*spa*-CCs) with Ridom Staph Type software [15]. Sequences containing less than 5 repeats were excluded from the analysis and *spa* types were clustered if the cost was less than or equal to 4, in order to prevent the formation of too large and non-specific *spa* clusters.

As *spa* types can unambiguously be mapped to the corresponding clonal lineages defined by multi locus sequence typing (MLST) to a large extent [12], we have also enlisted MLST based clonal complexes (MLST CC) according to the Ridom database (www.ridom.com).

The antibiotic susceptibility patterns of *S. aureus* isolates were checked for penicillin (susceptible ≤0.03), erythromycin (susceptible ≤0.25), ciprofloxacin (susceptible ≤0.5), tetracycline (susceptible ≤1), moxifloxacin (susceptible ≤0.25), oxacilin (susceptible ≤0.25), clindamycin (susceptible ≤0.25), gentamicin (susceptible ≤0.5), linezolid (susceptible ≤2), daptomycin (susceptible ≤0.25), vancomycin (susceptible ≤0.5), tigecycline (susceptible ≤0.12), fosfomycin (susceptible ≤8), fusidic acid (susceptible ≤0.5), rifampicin (susceptible ≤0.5) and trimethoprim/sulphamethoxazole (susceptible ≤10). The antibiotic susceptibility patterns were determined with the VITEK II system (AST-P611 card, bioMérieux, SA, Craponne, France) according to the manufacturer's instructions.

### Statistical analysis

In descriptive statistics, frequency and proportions were calculated for categorical variables and median and interquartile range (IQR) was reported for continuous variables. Possible determinants for *S. aureus* (including both MRSA and MSSA) nasal carriage were first checked through univariable logistic regression analysis. We applied multiple logistic regression by stepwise backward selection of variables with biological plausibility and a significance level of <0.10 for entry into the model. All statistical tests were considered significant with a p-value <0.05. Data were analyzed with IBM SPSS Statistics for Windows version 19.

### Ethics statement

The study was approved by the Ethics Committee of the State Board of Physicians of the German Federal State of Lower Saxony and the German Federal Commissioner for Data Protection and Freedom of Information (Approval letter numbers: Bo/13/2010 and 3137/2011). Written informed consent was obtained from all study participants.

## Results

### Characteristics of the sample

Among the 2026 invited persons, 405 (20%) agreed to participate and 389 (96%) of those participants sent a nasal swab along with the questionnaire during the period of July – September 2012. The study population included 237 (60.9%) female participants and the median age of the study population was 49 years (IQR: 39–61 years, range: 20–70 years). In the total of 389 participants, 276 (71.0%) visited outpatient clinics, 44 (11.3%) had been in the hospital and 158 (40.6%) were prescribed antibiotics, all in the last 12 months (Table 1).

**Table 1:** Descriptive characteristics of the study participants of the general population of Braunschweig, Germany.

Variables	Categories	N (%)
Sex	Female	237 (60.9)
	Male	152 (39.1)
Median age in years (inter quartile range)		49 (39–61)
Age	20–30 years	53 (13.6)
	31–40 years	51 (13.01)
	41–50 years	98 (25.2)
	51–60 years	89 (22.9)
	61–70 years	98 (25.2)
Allergies	No	182 (46.8)
	Yes	176 (45.2)
Diabetes mellitus	No	365 (93.8)
	Yes	16 (4.1)
Skin infection in the last 12 months	No	272 (69.9)
	Yes	104 (26.7)
Outpatient clinic visits in the last 12 months	No	110 (28.3)
	Yes	276 (71.0)
Hospital stay in the last 12 months	No	341 (87.7)
	Yes	44 (11.3)
Surgery in the last 12 months	No	339 (87.1)
	Yes	49 (12.6)
Prescribed antibiotics in the last 12 months	No	225 (57.8)
	Yes	158 (40.6)
Pet animal contact	No	185 (47.6)
	Yes	201 (51.7)
Occupational animals contact	No	377 (96.9)
	Yes	7 (1.8)
International travel in the last 12 months	No	162 (41.6)
	Yes	224 (57.6)

N = 389 (100%): Missing are included therefore percentage do not always add up to 100 percent.

### *S. aureus* nasal carriage prevalence and risk factors

*S. aureus* was detected in nasal swabs of 85 participants (21.9%; 95% confidence interval [CI]: 18.0–26.2%). MRSA was detected in five participants (1.29%; 95% CI: 0.55–2.98%), three of whom were female. The age of the five MRSA positive cases ranged from 28 to 64 years, and all five cases had a prior upper respiratory tract infection plus at least one of the following medical conditions: skin infection, gastrointestinal infection, herpes, allergies, or cardiovascular disease. Four of them had been prescribed antibiotics in the 12 months prior to the survey (Table 2).

**Table 2:** Profile of MRSA-positive cases identified in a random sample of 389 participants from the general population of Braunschweig, Germany.

Individual characteristics	Case 1	Case 2	Case 3	Case 4	Case 5
Age	28	49	56	64	29
Sex	male	female	female	female	male
Comorbidities	skin diseases, URTI	URTI, GIT, herpes, bladder infection	URTI, cardiovasculardiseases, allergies	URTI, LRTI, herpes, bladder infection	URTI, GIT
Family member working in health care	yes	no	no	no	yes
Family member working in child care	no	no	yes	no	no
Outpatient clinic visits in the last 12 months	yes	yes	yes	yes	yes
Hospital stay in the last 12 months	no	no	yes, operated in gynecology	no	no
Prescribed antibiotics in the last 12 months	yes	yes	yes	yes	no
Pet animal contact	cat	no	no	no	dog
International travel in the last 12 months	yes	no	yes	no	yes
*S. aureus spa* type	t032	t032	t025	t032	t032, *t330

Abbreviations: URTI; Upper respiratory tract infections, LRTI; Lower respiratory tract infections, GIT; Gastrointestinal tract infections.

*t330 was an MSSA.

In univariable logistic regression analysis, associations were observed between nasal *S. aureus* carriage and male sex (odds ratio [OR] = 2.66, 95% CI: 1.63–4.34) and presence of allergies (OR = 1.85, 95% CI: 1.10–3.09); no significant association was observed between nasal *S. aureus* carriage and pet animal contact, diagnosis of diabetes mellitus, occupational animal contact or outpatient clinic visits, prescribed antibiotics and international travel in the last 12 months (Table 3). In multiple logistic regression analysis, nasal carriage of *S. aureus* was also significantly associated with male sex (OR = 3.50, 95% CI: 2.01–6.11) and presence of allergies (OR = 2.43, 95% CI: 1.39–4.24).

**Table 3:** Univariable and multiple logistic regression analyses of factors associated with nasal *S. aureus* carriage among study participants of the general population of Braunschweig, Germany.

Variables	Categories	Univariable logistic regression OR (95% CI)	p-value	Multiple logistic regression OR (95% CI)	p-value
Sex	Female	1		1	
	Male	2.66 (1.63–4.34)	<0.001	3.50 (2.01–6.11)	<0.001
Age	61–70 years	1		1	
	20–30 years	2.14 (1.00–4.56)	0.049	2.52 (1.08–5.88)	0.085
	31–40 years	0.77 (0.31–1.91)	0.578	0.63 (0.23–1.71)	
	41–50 years	1.28 (0.64–2.53)	0.487	1.24 (0.59–2.64)	
	51–60 years	0.98 (0.47–2.03)	0.960	1.03 (0.46–2.29)	
Allergies	No	1		1	
	Yes	1.85 (1.10–3.09)	0.002	2.43 (1.39–4.24)	0.002
Diabetes mellitus	No	1			
	Yes	1.21 (0.38–3.84)	0.751		
Skin infection in the last 12 months	No	1			
	Yes	1.35 (0.79–2.29)	0.262		
Outpatient clinic visits in the last 12 months	No	1			
	Yes	0.94 (0.56–1.60)	0.833		
Hospital stay in the last 12 months	No	1			
	Yes	0.52 (0.21–1.28)	0.157		
Surgery in the last 12 months	No	1			
	Yes	1.04 (0.51–2.13)	0.922		
Prescribed antibiotics in the last 12 months	No	1			
	Yes	0.85 (0.51–1.39)	0.506		
Pet animal contact	No	1	0.423		
	Yes	0.82 (0.51–1.33)			
Occupational animal contact	No	1			
	Yes	1.44 (0.27–7.55)	0.667		
International travel in the last 12 months	No	1			
	Yes	1.43 (0.87–2.36)	0.159		

Abbreviations: OR; odds ratio, CI; confidence interval.

### Spa typing and BURP analysis

**Figure 1:**
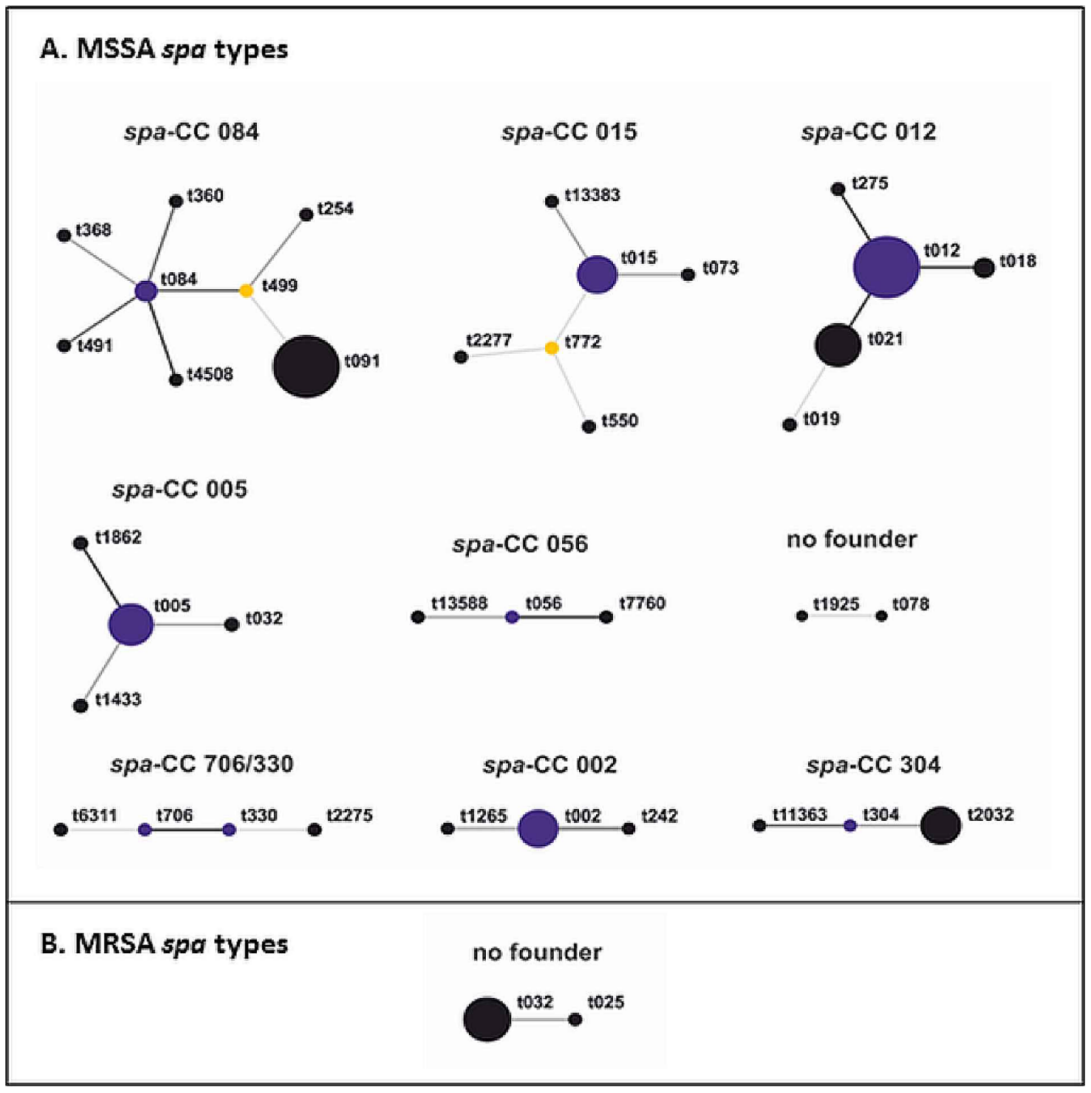
BURP analysis of the *S. aureus spa* types isolated from nasal swabs of 389 participants of the general population of Braunschweig, Germany. Each *spa* type identified is depicted with circles. Related *spa* types are connected with a black line; resultant clonal complexes are written in bold. The predicted founder of each clonal complex is indicated in blue and sub-founders are labelled in yellow. The size of the circle is proportional to the frequency of the *spa* type in the population. Each clonal complex (CC) is defined by the predicted founder *spa* type or by the *spa* types it contains.

Among the 85 isolates, fifty four different MSSA *spa* types were found, that clustered into nine distinct groups and two different MRSA *spa* types were clustered into one group (figure 1, table 4). Among the MSSA isolates, one cluster had no founder and 12 *spa* types (12/54, 22%) could not be clustered into a *spa*-CC and were classified as singletons. Six isolates (6/80, 8%) were excluded from the analysis as the X-region amplimer contained less than five repeats. Most MSSA *spa* types belonged to *spa*-CC 012 (16/80, 20%) and *spa*-CC 084 (15/80, 19%). Four of the MRSA belonged to *spa* type t032 and one to *spa* type t025 and had no founder (corresponds to MLST CC 22). From one sample, two different *spa* types, t032 (MRSA, *spa*-CC 005) and *spa* type t330 (MSSA, *spa*-CC 330/706) were isolated. MSSA *spa* types varied widely, 7 (9%) belong to *spa* types t012 (*spa*-CC 012, corresponds to MLST CC 30), 7 (9%) to *spa* types t091 (*spa*-CC 084, corresponds to MLST CC 15) and 5 (6%) were *spa* types t021 (*spa*-CC 012). One MSSA also had a *spa* type t032. We observed three new *spa* type's t13383, t13449 and t13588. One isolate was not typable (NT). Relatedness of the *spa* types belonging to the same clonal lineages identified by BURP analysis is shown in figure 1.

**Table 4:** Distribution of s*pa*-clonal complexes (*spa*-CCs) among isolates collected from nasal swabs of 389 participants of the general population of Braunschweig, Germany.

MSSA *spa* types				
*spa*-CC	*spa* types	Number of strains (% of all strains)	Number of *spa*-types (% of all *spa*-types)	MLST CC (ST)
*spa*-CC 084	t084, t091, t254, t360, t368, t491, t499, t4508	15 (19)	8 (15)	CC 15
*spa*-CC 015	t015, t073, t550, t772, t2277, t13383	7 (9)	6 (11)	CC 45
*spa*-CC 005	t005, t032, t1433, t1862	5 (6)	4 (7)	CC 22
*spa*-CC 012	t012, t018, t019, t021, t275	16 (20)	5 (9)	CC 30
*spa*-CC 706/330	t330, t706, t2275, t6311	4 (5)	4 (7)	
*spa*-CC 002	t002, t242, t1265	4 (5)	3 (6)	CC 5
*spa*-CC 304	t304, t2032, t11363	4 (5)	3 (6)	CC 8
*spa*-CC 056	t056, t7760, t13588	3 (4)	3 (6)	ST 101
No founder	t078, t1925	2 (3)	2 (4)	CC 45
Singletons	t159, t160, t537, t1057, t1541, t2313, t3741, t5337, t7088, t10983, t13449, t13587	14 (18)	12 (22)	
Excluded	t026, t132, t1977, t6115	6 (8)	4 (7)	

MRSA *spa* types				
*spa*-CC	*spa* types	Number of strains (% of all strains)	Number of *spa*-types (% of all *spa*-types)	MLST CC (ST)
No founder	t025, t032	5 (100)	2 (100)	CC 22

Total samples n = 85 (100%), MSSA (n = 80): 9 groups, 12 singletons, 4 *spa*-types excluded,

MRSA (n = 5): 1 group.

### Antimicrobial susceptibility testing

Table 5 provides antibiotic resistance patterns of the 85 *S. aureus* strains isolated from 389 study participants. Only 35 (41.2%) were sensitive to all tested antibiotics. Among the 85 isolates, resistance to penicillin was most common 49 (57.7%), and frequent resistance to fluoroquinolones 9 (10.6%) was also observed. All MRSA were resistant to penicillin and fluoroquinolones and one MRSA was also resistant to erythromycin and clindamycin.

**Table 5:** Antimicrobial resistance patterns of *S. aureus* isolated from the nasal swabs of the 389 participants of the general population of Braunschweig, Germany.

Antibiotic resistance	Frequency	Percent
Sensitive	35	41.2
PEN	38	44.7
PEN, CIP, MFL	2	2.4
PEN, CIP, MFL, ERY, CLI	1	1.2
PEN, ERY	1	1.2
PEN, ERY, CLI	1	1.2
PEN, OXA, CIP, MFL	4	4.7
PEN, OXA, CIP, MFL, ERY, CLI	1	1.2
PEN, TET	1	1.2
CIP, MFL	1	1.2
Total	85	100.0

Abbreviations: PEN: Penicillin, ERY: Erythromycin, CIP: Ciprofloxacin, TET: Tetracycline, MFL: Moxifloxacin, OXA: Oxacilin, CLI: Clindamycin.

All isolates tested were susceptible to vancomycin, tigecycline, gentamicin, linezolid, daptomycin, fosfomycin, fusidic acid, rifampicin and trimethoprim/sulphamethoxazole.

## Discussion

We determined the prevalence of MSSA and MRSA carriage in a random population-based study in one city of northern Germany. Every fifth participant in our study was colonized with *S. aureus*. In Europe, only two studies from the UK were based on random population-based samples, as is our study [4], [16]. Gamblin *et al* found 28% nasal *S. aureus* colonization in Southampton, which is close to a previous estimate of 23% by Abudu *et al* in Birmingham [4], [16]. Gamblin *et al* used a method similar to the one used in our study [4], [16]. The participants were requested to collect nasal swabs at home and return them by mail. However, the selection of participants from a single urban NHS general practice may have overestimated the prevalence in contrast to our study where a random sample was taken from the entire population of the city. We had a majority of female participants, which might lead to an underestimation of the prevalence detected in our study. In Italy and Norway, the prevalence of *S. aureus* is reported to be 25.9% and 27% respectively in the general community [3], [17]. In Italy, the study included those people who had visited university hospital for a voluntary check-up and in Norway nasal *S. aureus* carriage was checked in visitors of the two largest shopping centres in the region [3], [17]. The sampling approach in the Italian and Norwegian studies likely included an even stronger probability for recruiting bias, which might partly account for the higher *S. aureus* prevalence as compared to our study [3], [17]. In the US, only the studies by Gorwitz and Mainous were truly based on a random population based samples, as was the case in our study but resulted in higher S. *aureus* prevalence estimates [2], [18].

For the following reasons, we believe *S. aureus* prevalence assessed in this study to be an underestimate of the true population prevalence rather than an over estimate: (1) our study does not include high risk age groups such as children or people over 69 years of age (2) swabs from body sites other than anterior nares, such as pharyngeal and skin swabs, were not taken, (3) enrichment culture techniques were not used to enhance *S. aureus* detection.

We observed 1.3% MRSA prevalence in the community. This MRSA prevalence is lower than the previous estimate of MRSA colonization observed in one of the studies at the time of hospital admission in the state of Saarland, Germany [19] but quite similar to the estimates in the UK (1.1%), France (1.02%) and Italy (0.12%) taking the reported confidence intervals into account [3], [4], [20]. MRSA prevalence assessed in this study is not much different from other European studies and is slightly lower compared to the US (2.1%) as reported in one of the studies conducted on a large population-based random sample of 4666 adult participants [18]. MRSA prevalence appears to be significantly lower in Europe as compared to other parts of the world such as India (5.3%), China (3.6%) and Pakistan (2.8%) [21]–[23]. However, a high MRSA prevalence in these studies may be due to selection bias in the study design or less stringent antimicrobial treatment regimens and hygiene measures up to some extent.

Our finding that males were more likely to carry *S. aureus* is consistent with other studies [17], [18], [24], [25]. One of the recent population based studies conducted in Denmark on middle-aged and elderly twins also reported that men had a higher risk of *S. aureus* nasal carriage [24], indicating gender specific risk factors, not yet well understood, but also in line with observations from Norway, Denmark, Australia, New Zealand and the USA [17], [18], [24]–[28]. In contrast, more female carriers were observed in the National Health and Nutrition Examination Survey (2001) in the USA [2], [29]. Interestingly, a recent study in the South West of Germany found a significantly higher proportion of *S. aureus* colonization among females taking hormonal contraceptives [8]; this finding revives the discussion on hormonal disposition to *S. aureus* carriage. The literature is inconclusive with respect to differences in age [18], [24], [26]. Some studies found a higher prevalence in elderly people while others found a higher prevalence in younger adults.

Our findings confirm previously reported associations between allergies and *S. aureus* carriage [24], [25]. *S. aureus* toxins have been shown to accelerate allergic reactions in atopic diseases, asthma and allergic rhinitis, but a satisfactory mechanistic explanation for this observation has, so far, not been provided [24], [28], [30]–[31]. The other known risk factors could not be confirmed with significant associations in our study. Although the respective trends were visible, our study population most likely had insufficient power regarding less prevalent risk factors in the general population.

MRSA *spa* types t032 and t025 (MLST CC 22) represent the most frequent epidemic MRSA in German hospitals and are disseminated worldwide [32], [33], [34]. Spread of MRSA CC22 to the community was recently reported from countries with high prevalence of MRSA in hospitals [35], [36]. The high prevalence of particular clonal complexes observed in this study such as CC 15, CC30, and CC45 was also reported for isolates from nasal carriage by the study of Holtfreter *et al.*, performed in Germany in 2006 and also for isolates from the Netherlands, the USA and Portugal [7], [37], [38], [39]. These clonal complexes obviously have evolved as very successful colonizers of humans. The reasons for this success remain unknown so far. As nasal colonization is a main reservoir from which infections can start, it is no surprise to see isolates attributed to CC15 and to CC30 among the most frequent ones in a European study on *S. aureus* from invasive infections [40]. The multiple strain carriage was rare as only person was carrying MRSA *spa* type t032 and MSSA *spa* type t330.

More than half (60%) of the strains were resistant to one or more antibiotics and resistance to penicillin was most common. The MRSA isolates were also resistant to multiple antibiotics. Among MSSA isolates, the prevalence of resistance to fluoroquinolones, macrolides, aminoglycosides and other antibiotic classes (except penicillin) was still very low.

One of the limitations of this study is the possibility of selection bias. Although selection of the potential participant through the resident's registration office was random, response and participation in the study can be affected by age, sex and health related factors within the individuals. We had significantly more female participants (61%) as compared to the general population in Braunschweig (51%) [41]. Our response proportion of 20% is comparable with other studies based on invitation to participants through mail [4], [42]. However, the response proportion of the invitation accepting participants to send a nasal swab and the questionnaire was over 90%, which is also comparable with other studies [4], [42]. Use of self-swabbing may affect the quality of swab sampling. Sampling suitability was checked by the presence of microorganisms in general and only 3 (0.8%) had no bacterial growth [11]. Perfect agreement in terms of viral pathogen detection by PCR was observed in a previous study comparing staff-collected and participant-collected nasal swabs [43].

## Conclusion

This is the first study of *S. aureus* and MRSA point prevalence based on a random sample in a non-hospitalized population in Germany. It shows that *S. aureus* colonization in the Braunschweig population is similar to the reports available from other European countries. MRSA carriage is relatively uncommon in this community. The detection of hospital-associated MRSA *spa* types in the community indicate possible spread of these strains from hospitals into the community. Given the positive experience with self-sampling in a general population based design, a larger point prevalence study, including children and elderly people might help to establish risk factors for MRSA in the general population, which would in turn contribute relevant evidence for screening algorithms and other prevention methods.
